# The endothelial activation and stress index is a potential prognostic indicator for patients with acute pancreatitis managed in the intensive care unit: a retrospective study

**DOI:** 10.3389/fmed.2024.1498148

**Published:** 2024-12-11

**Authors:** Jianjun Wang, Xi Chen, Chuan Qin, Xintao Zeng, Xiaobo Du, Decai Wang

**Affiliations:** ^1^Department of Hepatobiliary Surgery, Mianyang Central Hospital, School of Medicine, University of Electronic Science and Technology of China, Mianyang, China; ^2^NHC Key Laboratory of Nuclear Technology Medical Transformation, Mianyang Central Hospital, School of Medicine, University of Electronic Science and Technology of China, Mianyang, China; ^3^Department of Oncology, Mianyang Central Hospital, School of Medicine, University of Electronic Science and Technology of China, Mianyang, China; ^4^Department of Urology, Mianyang Central Hospital, School of Medicine, University of Electronic Science and Technology of China, Mianyang, China

**Keywords:** acute pancreatitis, EASIX, all-cause mortality, endothelial dysfunction, MIMIC-IV

## Abstract

**Background:**

The endothelial activation and stress index (EASIX) serves as a dependable and efficient surrogate marker for endothelial dysfunction, which plays an essential role in the pathophysiology of acute pancreatitis (AP). Hence, we investigated the prognostic value of EASIX in AP.

**Methods:**

This was a retrospective study, using patient information obtained from the Medical Information Market for Intensive Care-IV (MIMIC-IV) database. EASIX was calculated using lactate dehydrogenase, serum creatinine, and platelet counts obtained during the first measurement within 24 h of admission. Patients were grouped into three cohorts based on log2-transformed EASIX. The main endpoint of the study was 28-day all-cause mortality (ACM) in AP patients, with the secondary endpoint being 90-day ACM. The relationship between EASIX and prognosis in patients with AP was evaluated using Cox proportional hazards models, Kaplan–Meier curves, restricted cubic spline (RCS) curves, and subgroup analyses. Receiver operating characteristic (ROC) curves were constructed to evaluate the predictive performance of EASIX compared to other indicators.

**Results:**

The study cohort comprised 620 patients in total. Multivariate Cox proportional hazards analysis indicated that an increased log2 (EASIX) was linked to a higher risk of 28-day ACM in AP patients (HR, 1.32; 95% CI: 1.14–1.52; *p* < 0.001). The risk of 28-day ACM was higher in Tertiles 2 and 3 compared with Tertile 1 [(HR, 2.80; 95% CI: 1.21–6.45); (HR, 3.50; 95% CI: 1.42–8.66)]. Comparable findings were noted for 90-day ACM. Kaplan–Meier curves demonstrated that patients with elevated log2 (EASIX) had lower 28- and 90-day survival rates. The RCS curves suggested a non-linear relationship between log2 (EASIX) and 28- and 90-day ACM. ROC curves indicated that log2 (EASIX) was not inferior to sequential organ failure assessment and systemic inflammatory response syndrome scores in predicting the prognosis of patients with AP. Subgroup analyses demonstrated no interaction between log2 (EASIX) and any subgroup.

**Conclusion:**

Elevated EASIX levels were significantly correlated with a heightened risk of 28- and 90-day ACM in AP patients.

## 1 Introduction

Acute pancreatitis (AP) is a prevalent digestive system disease characterized by a spectrum of clinical presentations, from mild, self-limiting episodes to severe complications and high mortality rates (1). Epidemiological investigations have shown that the annual incidence of AP is approximately 13–45 cases per 100,000 individuals (2). Cholelithiasis, alcohol abuse, and metabolic disorders (such as hyperlipidemia, hypercalcemia, and hyperuricemia) are common causes of AP (3). Furthermore, AP can be induced or aggravated by pancreatic tumors, pancreatic duct stones, certain drugs, and viral infections (3). Approximately 80% of patients with AP suffer from mild symptoms, which can be cured by fasting, rehydration, nutritional support, and other treatments, while 20% of patients are in severe condition, which manifests as necrosis of the pancreas and peripancreatic tissues, and even multi-organ failure. The mortality rate of these patients is as high as 20–40% (4, 5). Therefore, accurately evaluating the severity of AP and providing early intervention for critically ill patients holds great clinical significance.

The endothelial activation and stress index (EASIX) is identified as an innovative biomarker for endothelial dysfunction and inflammatory response, and is calculated from routine laboratory parameters, including lactate dehydrogenase (LDH), serum creatinine (Crea), and platelet counts (PLT) (6). In 2017, Luft et al. (6) reported for the first time that EASIX has the potential to predict survival outcomes in patients with acute graft-versus-host disease following allogeneic hematopoietic stem cell transplantation. Subsequently, EASIX has been demonstrated to have a close prognostic association with a variety of oncological and non-oncological diseases, such as multiple myeloma, novel coronavirus pneumonia, diffuse large B-cell lymphoma, small-cell lung cancer, and sepsis (7–11).

Endothelial dysfunction is a key factor in the pathophysiology of AP, which promotes pancreatic and other organ damage through such mechanisms as inflammatory responses, microcirculatory disturbances, vasoactive substance imbalance, and apoptosis of vascular endothelial cells (12–14). More importantly, laboratory parameters that comprise EASIX, including LDH, Crea, and PLT, have been demonstrated to be important prognostic factors in AP (15–17). Given that the association between EASIX and the prognosis of patients with AP has not been previously explored, our research team aimed to investigate this relationship in AP patients managed in the intensive care unit (ICU) using data from the Medical Information Mart for Intensive Care-IV (MIMIC-IV, version 2.2) database. Specifically, we sought to assess the relationship between EASIX and 28- and 90-day all-cause mortality (ACM) in patients with AP after admission and assess its predictive value. By elucidating the prognostic significance of EASIX in AP, our study may contribute to the early risk stratification of AP and optimization of clinical management strategies.

## 2 Methods

### 2.1 Source of data

Patient data for this study were sourced exclusively from the MIMIC-IV (v 2.2) database, a comprehensive and publicly accessible resource maintained by the Laboratory of Computational Physiology at the Massachusetts Institute of Technology (18). It includes inpatient data for all patients admitted to the Beth Israel Deaconess Medical Center (BIDMC). To ensure patient privacy, all personal information was de-identified, with random codes used to substitute for patient identifiers. Therefore, BIDMC waived the need for informed consent and ethical approval. Our research team completed all the courses of the Collaborative Institutional Training Initiative for the MIMIC-IV (v 2.2) database, passed the “Conflict of Interest” and “Study Data or Specimens Only” exams, and was granted access.

### 2.2 Study population

**Figure 1:**
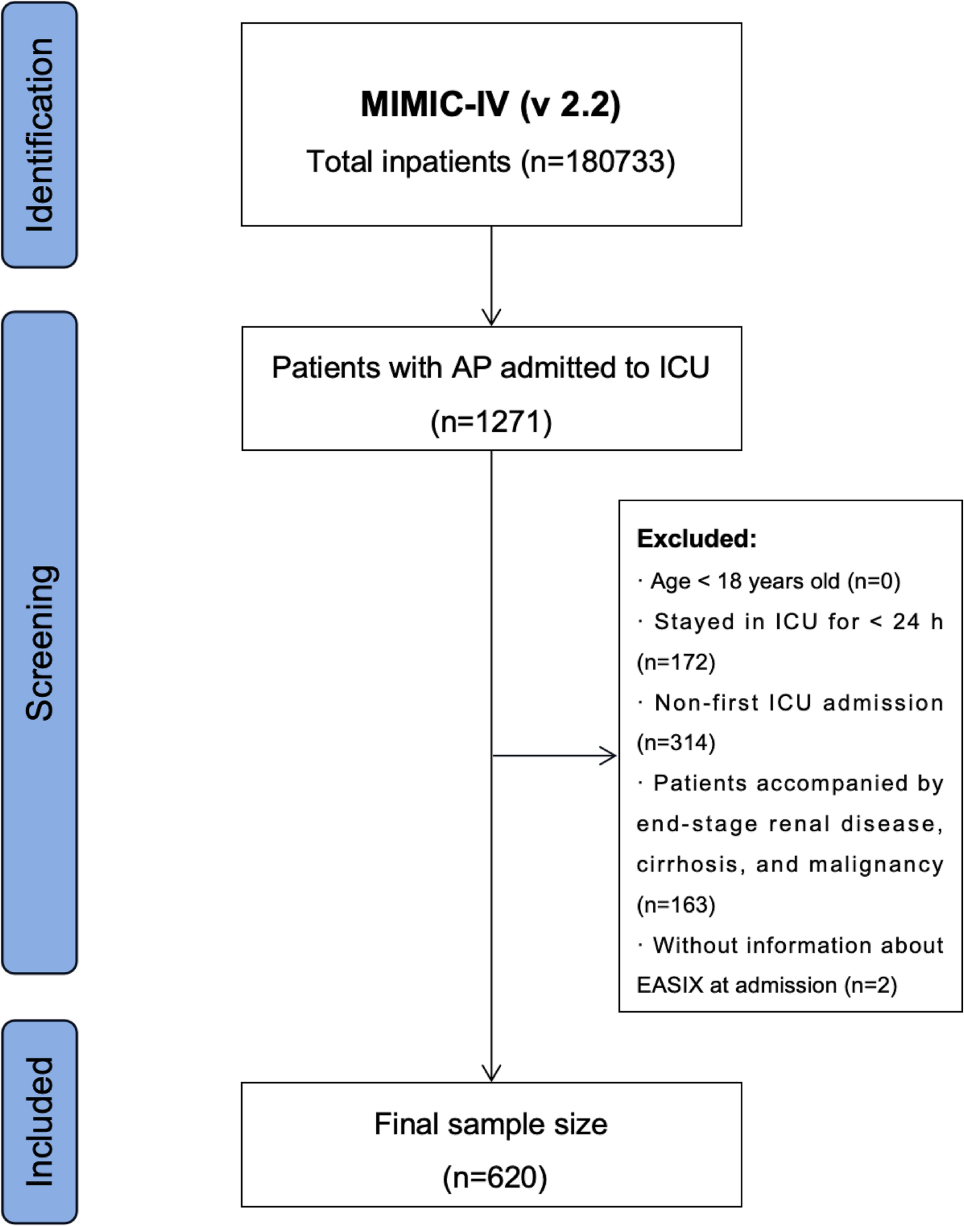
Flowchart of the patient selection process.

Data regarding the hospitalization of patients with AP were extracted using the International Classification of Diseases, Ninth Revision (ICD-9) code 577.0, and Tenth Revision (ICD-10) codes K85-K85.92. Stringent exclusion criteria were established based on the study objectives: (1) patients younger than 18 years at their initial admission; (2) patients with recurrent ICU admissions for AP; (3) patients with end-stage renal disease, cirrhosis, or malignancy; (4) patients with ICU stays of less than 24 h; and (5) patients with missing data on LDH, Crea, and PLT within the first 24 h post-admission. A total of 620 patients were enrolled in this study (Figure 1).

### 2.3 Extraction of covariates

Covariate extraction was performed using structured query language (SQL). The covariate extraction tools included PostGresSQL (version 13.7.2) and Navicate Premium (version 16). The covariates extracted in this study comprised five main components: demographic characteristics, vital signs, clinical treatments, comorbidities, and laboratory variables. Additional details are listed in Table 1. EASIX was calculated by the formula: LDH (U/L) × Crea (mg/dL) / PLT (109 cells/L). Remarkably, we used a variety of validation methods to ensure the accuracy of the extracted covariates, such as an independent review and consistency check of key data points and the use of appropriate statistical software to identify and correct possible input errors.

**Table 1:** Covariates extracted from MIMIC-IV (v2.2).

Items	Composition
Demographic variables	Age, Gender, Ethnicity
Vital signs	HR, SBP, DBP, MAP, RR, SpO2, Temperature
Clinical treatments	Vasopressin, Octreotide, Statins, Betablockers, Ventilation, CRRT, ERCP
Comorbidities	AKI, Sepsis, RF, HF, AF, Hypertension, Diabetes, Obesity
Laboratory variables	RBC, WBC, RDW, PLT, Hb, NEUT, HCT, AG, ALB, TBil, AST, ALT, GLU, Crea, BUN, PT, INR, K, Na, Ca, LAC, Blood lipase, Blood amylase
Clinical outcomes	28-day ACM, 90-day ACM

HR, heart rate; SBP, systolic blood pressure; DBP, diastolic blood pressure; MAP, mean arterial pressure; RR, respiratory rate; CRRT, continuous renal replacement therapy; ERCP, endoscopic retrograde cholangiopancreatography; AKI, acute kidney injury; RF, respiratory failure; HF, heart failure; AF, atrial fibrillation; RBC, red blood cell; WBC, white blood cell; RDW, erythrocyte distribution width; PLT, platelet; Hb, hemoglobin; NEUT, neutrophil count; HCT, hematocrit; AG, anion gap; ALB, albumin; TBil, total bilirubin; AST, aspartate aminotransferase; ALT, alanine aminotransferase; GLU, glucose; Crea, creatinine; BUN, blood urea nitrogen; PT, prothrombin time; INR, international normalized ratio; K, serum potassium; Na, serum sodium; Ca, serum total calcium; LAC, lactate; ACM, all-cause mortality.

### 2.4 Handling of abnormal and missing values

Using the STATA winsor2 command, the outlier variables were processed using the winsorization method at 1 and 99% cut-offs. Covariates with more than 15% missing data, such as C-reactive protein and calcitonin levels, were excluded.

### 2.5 Study endpoints

The primary and secondary endpoints of this study were 28- and 90-day ACM after admission, respectively.

### 2.6 Statistical analysis

Categorical variables are expressed as numbers (percentages) and were compared using either a chi-square test or Fisher’s exact test. Continuous variables with a normal distribution are reported as means ± standard deviations, while those with a skewed distribution are presented as medians with interquartile ranges. Normally distributed continuous variables were analyzed using a t-test or ANOVA, whereas the Mann–Whitney U-test or Kruskal-Wallis test was applied for skewed distributions. Because of the skewed distribution of EASIX, it was log-transformed prior to analysis.

Kaplan–Meier survival analyses were performed to assess and compare the distribution and differences in 28- and 90-day ACM after admission in different groups of patients with AP. Cox proportional hazards models were employed to evaluate the relationship between log2 (EASIX) and 28- and 90-day ACM in AP patients. Hazard ratios (HRs) and 95% confidence intervals (CIs) were calculated, with adjustments for potential confounders in various models. Model 1 was adjusted for no covariates. Model 2 was adjusted for age, sex, and ethnicity. Model 3 was additionally adjusted for systolic blood pressure (SBP), diastolic blood pressure (DBP), mean arterial pressure (MAP), red blood cells (RBC), white blood cells (WBC), PLT, hemoglobin (Hb), aspartate aminotransferase (AST), alanine aminotransferase (ALT), hypertension, diabetes, heart failure (HF), atrial fibrillation (AF), respiratory failure (RF), and obesity. After grouping patients according to tertiles, Tertile 1 was selected as the reference group and calculated adjusted HRs for the 28- and 90-day ACM in other groups. Restricted cubic spline (RCS) analysis was conducted to investigate the non-linear relationship between log2 (EASIX) and 28- and 90-day ACM post-admission in AP patients. Receiver operating characteristic (ROC) analysis was utilized to evaluate the predictive capability of log2 (EASIX) for 28- and 90-day ACM, with the area under the curve (AUC) being calculated. Sequential organ failure assessment (SOFA) and systemic inflammatory response syndrome (SIRS) scores have well-recognized utility in assessing the prognosis of critically ill patients with AP (2). SOFA is widely recognized for assessing organ dysfunction and predicting prognosis in the ICU setting, and it reflects the severity of multiple organ failure-a key determinant of AP prognosis. Similarly, the SIRS score is frequently used in the assessment of AP because it recognizes the systemic inflammatory response, which is strongly associated with the prognosis of AP. Therefore, we compared log2 (EASIX) with SOFA and SIRS scores in assessing the prognosis of AP patients. Finally, subgroup analyses were performed to investigate whether the prognostic value of log2 (EASIX) was consistent across subgroups. These subgroups were based on age, sex, hypertension, diabetes, and RF levels. The software used for the data analysis included R (version 4.2.2), STATA (version 16.0), and IBM SPSS (version 22.0). A two-tailed significance threshold of *p* < 0.05 (two-tailed) was required for all analyses.

## 3 Results

### 3.1 Baseline demographic and clinical characteristics

In total, 620 patients were included in this study. The baseline characteristics of the patients are presented in Table 2. The median age of participants was 57 (44–71) years, and 57.74% were male. In patients with a higher log2 (EASIX), the incidence of acute kidney injury, sepsis, RF, heart failure, and atrial fibrillation was also relatively higher. Patients with higher log2 (EASIX) also had higher SOFA scores. In addition, the use of vasopressin, ventilation, and continuous renal replacement therapy was higher in patients with higher log2 (EASIX). With increasing log2 (EASIX), erythrocyte distribution width, neutrophil counts, anion gap, total bilirubin, AST, Crea, blood urea nitrogen, serum potassium, lactate, blood lipase, and blood amylase levels increased, whereas PLT and serum calcium levels decreased. Our findings showed that the 28- and 90-day ACM rates in patients with AP were 11.29 and 17.9%, respectively. Patients with higher log2 (EASIX) also exhibited higher 28- and 90-day ACM (All *p* < 0.001). The detailed results are presented in Table 2.

**Table 2:** Baseline characteristics of participants.

Variables	Overall (*n* = 620)	Tertile 1 (*n* = 206)	Tertile 2 (*n* = 207)	Tertile 3 (*n* = 207)	*p* value
log2 (EASIX)	0.96 (−0.05–2.44)	−0.56 (−1.16 to −0.05)	0.96 (0.66–1.36)	3.25 (2.44–4.38)	
Demographics					
Age, years	57 (44–71)	54 (44–68)	61 (44–76)	59 (45–69)	0.02
Men, *n* (%)	358 (57.74)	101 (49.03)	120 (57.97)	137 (66.18)	0.002
Ethnicity, *n* (%)					
Asian populations	20 (3.23)	3 (1.46)	6 (2.90)	11 (5.31)	0.16
White populations	386 (62.26)	136 (66.02)	124 (59.90)	126 (60.87)	
Black populations	57 (9.19)	18 (8.74)	25 (12.08)	14 (6.76)	
Others	157 (25.32)	49 (23.79)	52 (25.12)	56 (27.05)	
Cause of AP, *n* (%)					
Biliary-related AP	42 (6.77)	8 (3.88)	23 (11.11)	11 (5.31)	0.005
Alcohol-related AP	50 (8.06)	12 (5.83)	16 (7.73)	22 (10.63)	
Drug-related AP	5 (0.81)	0 (0.00)	1 (0.48)	4 (1.93)	
Unknown	523 (84.35)	186 (90.29)	167 (80.68)	170 (82.13)	
Comorbidities					
AKI, n (%)	436 (70.32)	129 (62.62)	135 (65.22)	172 (83.09)	<0.001
Sepsis, n (%)	456 (73.55)	125 (60.68)	156 (75.36)	175 (84.54)	<0.001
RF, n (%)	283 (45.65)	72 (34.95)	88 (42.51)	123 (59.42)	<0.001
HF, n (%)	105 (16.94)	26 (12.62)	46 (22.22)	33 (15.94)	0.03
AF, n (%)	133 (21.45)	32 (15.53)	53 (25.60)	48 (23.19)	0.03
Hypertension, n (%)	296 (47.74)	107 (51.94)	108 (52.17)	81 (39.13)	0.01
Diabetes mellitus, n (%)	201 (32.42)	55 (26.70)	75 (36.23)	71 (34.30)	0.09
Obesity, n (%)	82 (13.23)	31 (15.05)	25 (12.08)	26 (12.56)	0.63
Vital signs					
HR, beats/min	101(85–117)	104 (89–120)	97 (82–116)	100 (83–115)	0.01
SBP, mmHg	126 (110–145)	131 (114–148)	130 (110–148)	122 (101–142)	0.002
DBP, mmHg	72(60–87)	75 (65–88)	73 (60–89)	67 (56–82)	<0.001
MAP, mmHg	91.0 (78.3–104.5)	94.0 (83.0–106.0)	92.3 (79.3–108.0)	86 (73–99.7)	<0.001
RR, times/min	21(17–25)	20 (17.5–26)	21(16–26)	21 (17–25)	0.99
SPO_2_, %	96 (94–99)	96 (94–99)	96 (94–99)	97 (94–99)	0.77
Temperature, °C	36.8 (36.4–37.3)	36.9 (36.6–37.5)	36.8 (36.4–37.3)	36.7 (36.3–37.3)	0.34
Laboratory parameters					
RBC, 10^9^/L	3.62 (3.12–4.19)	3.56 (3.10–4.15)	3.74 (3.28–4.26)	3.52 (3.00–4.15)	0.02
WBC, 10^9^/L	12.8 (9.0–18.4)	13.8 (9.7–18.7)	13.1 (9.1–18.1)	11.9 (7.5–18.1)	0.08
RDW, %	14.5 (13.6–15.8)	14.4 (13.6–15.6)	14.5 (13.5–15.7)	14.7 (13.9–16.3)	0.01
PLT, 10^9^/L	185 (128.5–261.5)	272 (199–365)	182 (136–233)	126 (84–173)	<0.001
Hb, g/L	11.1 (9.6–12.7)	10.8 (9.4–12.2)	11.4 (10.1–13.1)	11 (9.6–13)	0.01
NEUT, 10^9^/L	8.39 (5.09–13.98)	7.83 (4.58–14.24)	7.91 (4.86–13.00)	9.07 (5.97–14.53)	0.34
HCT	33.4 (29.0–38.4)	32.7 (28.7–36.9)	34.3 (30.2–39.1)	33.2 (28.8–39.5)	0.03
AG, mmol/L	14 (12–17)	13 (12–15)	14 (12–16)	16 (13–20)	<0.001
ALB, g/dL	2.9 (2.5–3.3)	2.9 (2.4–3.3)	3.0 (2.5–3.4)	2.9 (2.5–3.3)	0.14
TBil, mg/dL	1.1 (0.6–2.9)	0.7 (0.5–1.5)	1.2 (0.6–3.1)	1.6 (0.7–4.1)	<0.001
ALT, U/L	54 (24–129)	34.5 (17–75)	75.5 (29.5–145)	60 (30–192)	<0.001
AST, U/L	67 (34–166)	40.5 (25–80)	77 (41–179)	110 (50–290)	<0.001
GLU, mg/dL	130 (103–176)	125 (102–162)	134 (108–185)	131.5 (97–186)	0.06
Crea, mg/dL	1.1 (0.7–1.8)	0.7 (0.5–0.9)	1.1 (0.8–1.5)	2.1 (1.4–4.0)	<0.001
BUN, mg/dL	21 (12–35)	13 (9–18)	21 (13–32)	37 (23–57)	<0.001
PT, s	14.4 (12.9–16.8)	14.3 (13–16)	14.1 (12.9–16.2)	14.9 (12.9–18.3)	0.03
INR	1.3 (1.2–1.5)	1.3 (1.2–1.5)	1.3 (1.2–1.5)	1.3 (1.2–1.7)	0.07
K, mmol/L	4.0 (3.6–4.6)	3.9 (3.6–4.3)	4.0 (3.6–4.6)	4.3 (3.7–4.9)	<0.001
Na, mmol/L	138 (135–141)	138 (135–141)	139 (136–142)	138 (134–142)	0.02
Ca, mg/dL	7.9 (7.3–8.4)	8.0 (7.6–8.5)	8.0 (7.2–8.5)	7.6 (6.9–8.3)	<0.001
LAC, mmol/L	1.8 (1.2–2.8)	1.5 (1.1–2.4)	1.8 (1.3–2.6)	2.2 (1.4–3.4)	<0.001
Blood lipase, U/L	256 (62–1,164)	122.5 (40.5–600)	290 (60–1,415)	468 (91–1,509)	<0.001
Blood amylase, U/L	140 (58–376)	100 (51–266)	135 (59–404)	194 (65–534)	0.002
Score					
SOFA	1 (0–3)	0 (0–1)	1 (0–3)	2 (0–5)	<0.001
Treatments					
Vasopressin, *n* (%)	93 (15.00)	16 (7.77)	25 (12.08)	52 (25.12)	<0.001
Octreotide, *n* (%)	60 (9.68)	20 (9.71)	17 (8.21)	23 (11.11)	0.61
Statins, *n* (%)	198 (31.94)	61 (29.61)	78 (37.68)	59 (28.50)	0.09
Betablockers, *n* (%)	365 (58.87)	120 (58.25)	127 (61.35)	118 (57.00)	0.65
Ventilation, *n* (%)	544 (87.74)	176 (85.44)	176 (85.02)	192 (92.75)	0.03
CRRT, *n* (%)	81 (13.06)	3 (1.46)	17 (8.21)	61 (29.47)	<0.001
ERCP, *n* (%)	40 (6.45)	18 (8.74)	14 (6.76)	8 (3.86)	0.13
Clinical outcomes					
28-day ACM, *n* (%)	70 (11.29)	9 (4.369)	24 (11.59)	37 (17.87)	<0.001
90-day ACM, *n* (%)	111 (17.90)	22 (10.68)	36 (17.39)	53 (25.60)	<0.001

EASIX, endothelial activation and stress index; AP, acute pancreatitis; AKI, acute kidney injury; RF, respiratory failure; HF, heart failure; AF, atrial fibrillation; HR, heart rate; SBP, systolic blood pressure; DBP, diastolic blood pressure; MAP, mean arterial pressure; RR, respiratory rate; RBC, red blood cell; WBC, white blood cell; RDW, erythrocyte distribution width; PLT, platelet; Hb, hemoglobin; NEUT, neutrophil count; HCT, hematocrit; AG, anion gap; ALB, albumin; TBil, total bilirubin; AST, aspartate aminotransferase; ALT, alanine aminotransferase; GLU, glucose; Crea, creatinine; BUN, blood urea nitrogen; PT, prothrombin time; INR, international normalized ratio; K, serum potassium; Na, serum sodium; Ca, serum total calcium; LAC, lactate; SOFA, Sequential Organ Failure Assessment; CRRT, continuous renal replacement therapy; ERCP, endoscopic retrograde cholangiopancreatography; ACM, all-cause mortality.

### 3.2 Kaplan–Meier survival curve analysis

**Figure 2:**
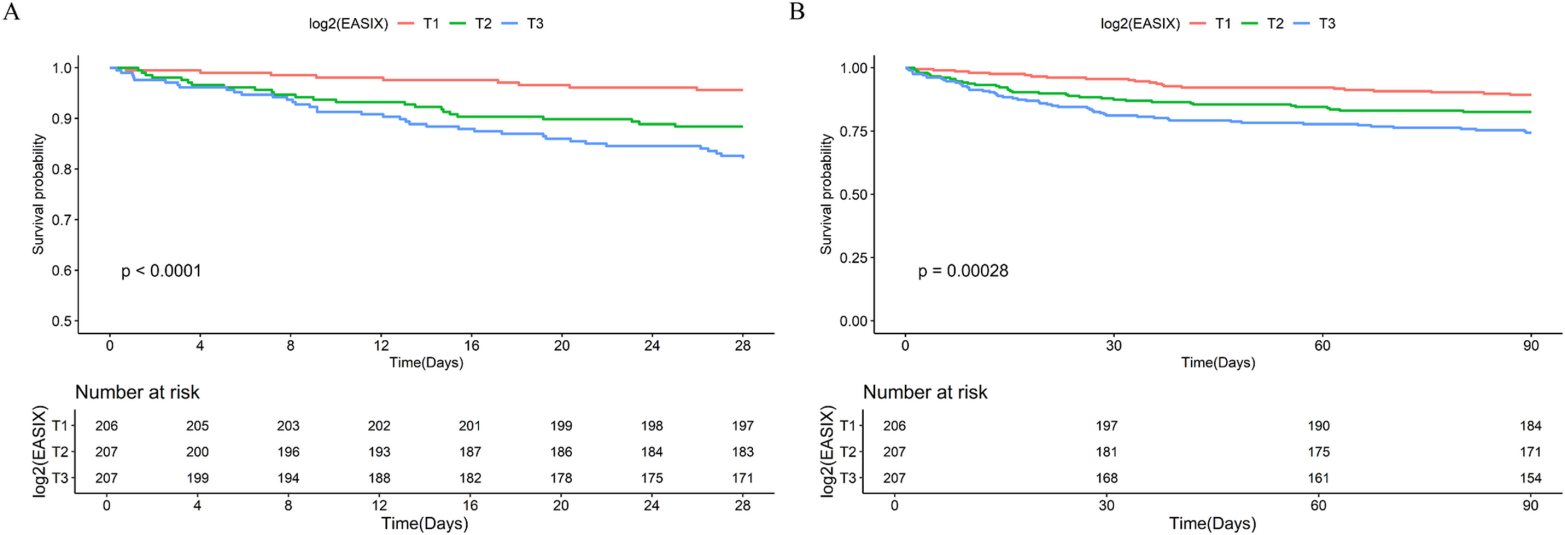
Kaplan–Meier survival analysis curves for all-cause mortality (ACM). Kaplan–Meier curves and cumulative incidence of 28- **(A)** and 90-day **(B)** ACM stratified by log2 (EASIX) tertiles.

To further explore the relationship between log2 (EASIX) and 28- and 90-day ACM in patients with AP, we plotted Kaplan–Meier cumulative curves (Figure 2). Patients with a higher log2 (EASIX) also had higher 28-and 90-day ACM (*p* for the log-rank test, <0.0001 and 0.00028, respectively).

### 3.3 Association of log2 (EASIX) with 28- and 90-day ACM in patients with AP

Cox proportional hazards regression models were developed to evaluate the relationship between log2 (EASIX) and the prognosis of patients with AP (Table 3). The results indicated that log2 (EASIX) was positively associated with 28-day ACM in patients with AP. In Model 1 with unadjusted covariates, the HRs (95% CI) for Tertiles 2 and 3 in relation to log2 (EASIX), were 2.78 (95% CI: 1.29–5.97) and 4.39 (95% CI: 2.12–9.09), respectively. In Model 2, after adjusting for age, sex, and ethnicity, the HRs (95% CI) for Tertiles 2 and 3 were 2.50 (95% CI: 1.16–5.40) and 4.15 (95% CI: 1.98–8.70), respectively. When additionally adjusted for SBP, DBP, MAP, RBC, WBC, PLT, Hb, AST, ALT, hypertension, diabetes, HF, AF, RF, and obesity, a significant positive correlation remained. In Model 3, adjusted HRs (95% CI) were 2.80 (95% CI: 1.21–6.45) and 3.50 (95% CI: 1.42–8.66) for Tertiles 2 and 3, respectively. Analyzing log2 (EASIX) as a continuous variable revealed a significant positive correlation with 28-day ACM in both unadjusted and adjusted models.

**Table 3:** Cox proportional hazard ratios (HR) for ACM.

	Model 1		Model 2		Model 3	
	HR (95% CI)	*p*- value	HR (95% CI)	*p*- value	HR (95% CI)	*p*- value
EASIX^*^ (continuous)	1.29 (1.17–1.42)	<0.001	1.35 (1.20–1.50)	<0.001	1.32 (1.14–1.52)	<0.001
EASIX^*^ (tertiles)						
Tertile 1	Reference		Reference		Reference	
Tertile 2	2.78 (1.29–5.97)	0.009	2.50 (1.16–5.40)	0.02	2.80 (1.21–6.45)	0.02
Tertile 3	4.39 (2.12–9.09)	<0.001	4.15 (1.98–8.70)	<0.001	3.50 (1.42–8.66)	0.007
*P* for trend	<0.001		<0.001		0.01	
90-day ACM						
EASIX^*^ (continuous)	1.21 (1.12–1.32)	<0.001	1.27 (1.16–1.39)	<0.001	1.27 (1.14–1.42)	<0.001
EASIX^*^ (tertiles)						
Tertile 1	Reference		Reference		Reference	
Tertile 2	1.73 (1.02–2.94)	0.04	1.50 (0.88–2.57)	0.14	1.86 (1.04–3.34)	0.04
Tertile 3	2.66 (1.62–4.38)	<0.001	2.64 (1.59–4.39)	<0.001	2.63 (1.39–4.97)	0.003
*P* for trend	<0.001		<0.001		0.003	

*Log2 (EASIX). Model 1: Unadjusted; Model 2: Adjusted for age, gender, and ethnicity; Model 3: Model 2+ additionally adjusted for systolic blood pressure, diastolic blood pressure, mean arterial pressure, red blood cell, white blood cell, platelet, hemoglobin, aspartate aminotransferase, alanine aminotransferase, hypertension, diabetes, heart failure, atrial fibrillation, respiratory failure, and obesity.

Comparable findings were obtained when evaluating the relationship between log2 (EASIX) and 90-day ACM using Cox proportional hazards regression models. Additional details are presented in Table 3.

### 3.4 Detection of a non-linear relationship

**Figure 3:**
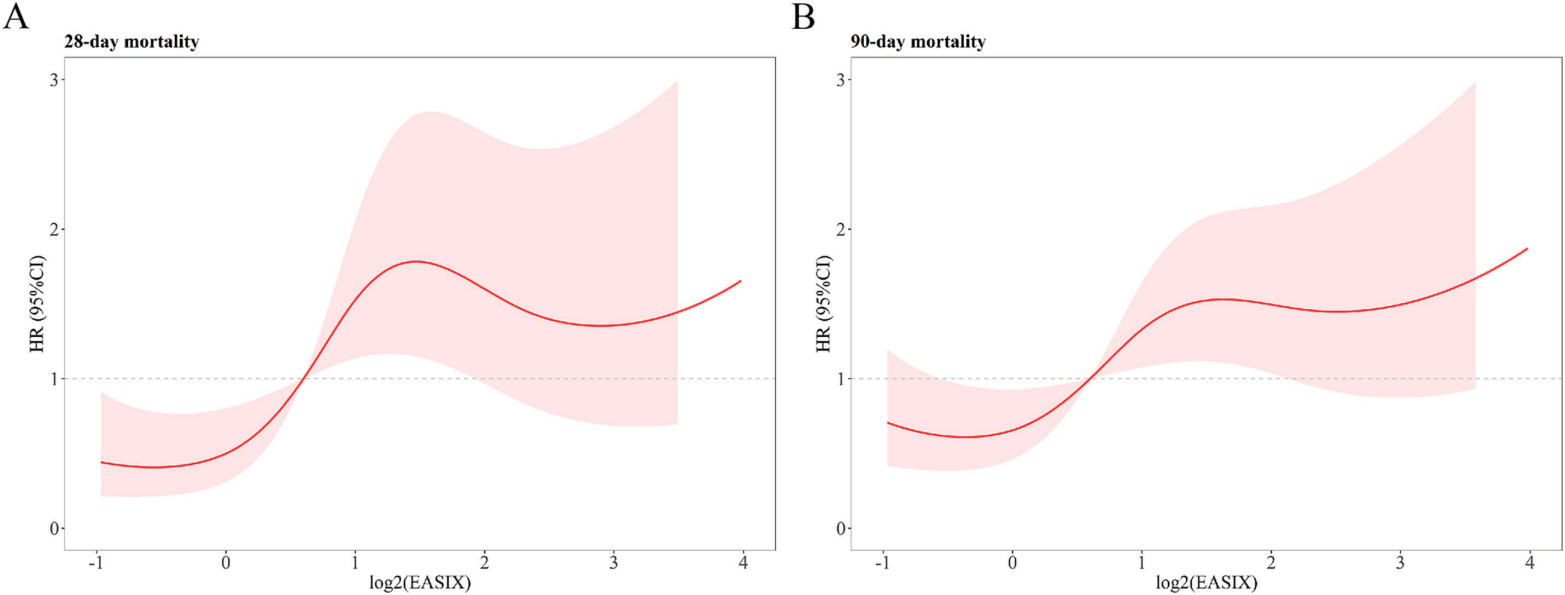
Restricted cubic spline analysis showing the relationship of log2 (EASIX) with 28- **(A)** and 90-day **(B)** all-cause mortality (ACM) in patients with acute pancreatitis (AP). 28-day ACM: *P* for no-linear = 0.047. 90-day ACM: *P* for no-linear = 0.03.

To further analyze the non-linear relationship of log2 (EASIX) with 28- and 90-day ACM, RCS regression was performed. For 28-day ACM, the HR increased and then decreased with increasing log2 (EASIX) and eventually increased again at higher log2 (EASIX) values (*p* = 0.047; Figure 3A). For 90-day ACM, the HR similarly increased first with increasing log2 (EASIX), then decreased slightly in the middle part, and finally increased again (*p* = 0.03; Figure 3B). These results suggest that log2 (EASIX) is a significant predictor of 28- and 90-day ACM, and that the relationship is predominantly non-linear.

### 3.5 Prediction of ACM in patients with AP using log2 (EASIX)

**Figure 4:**
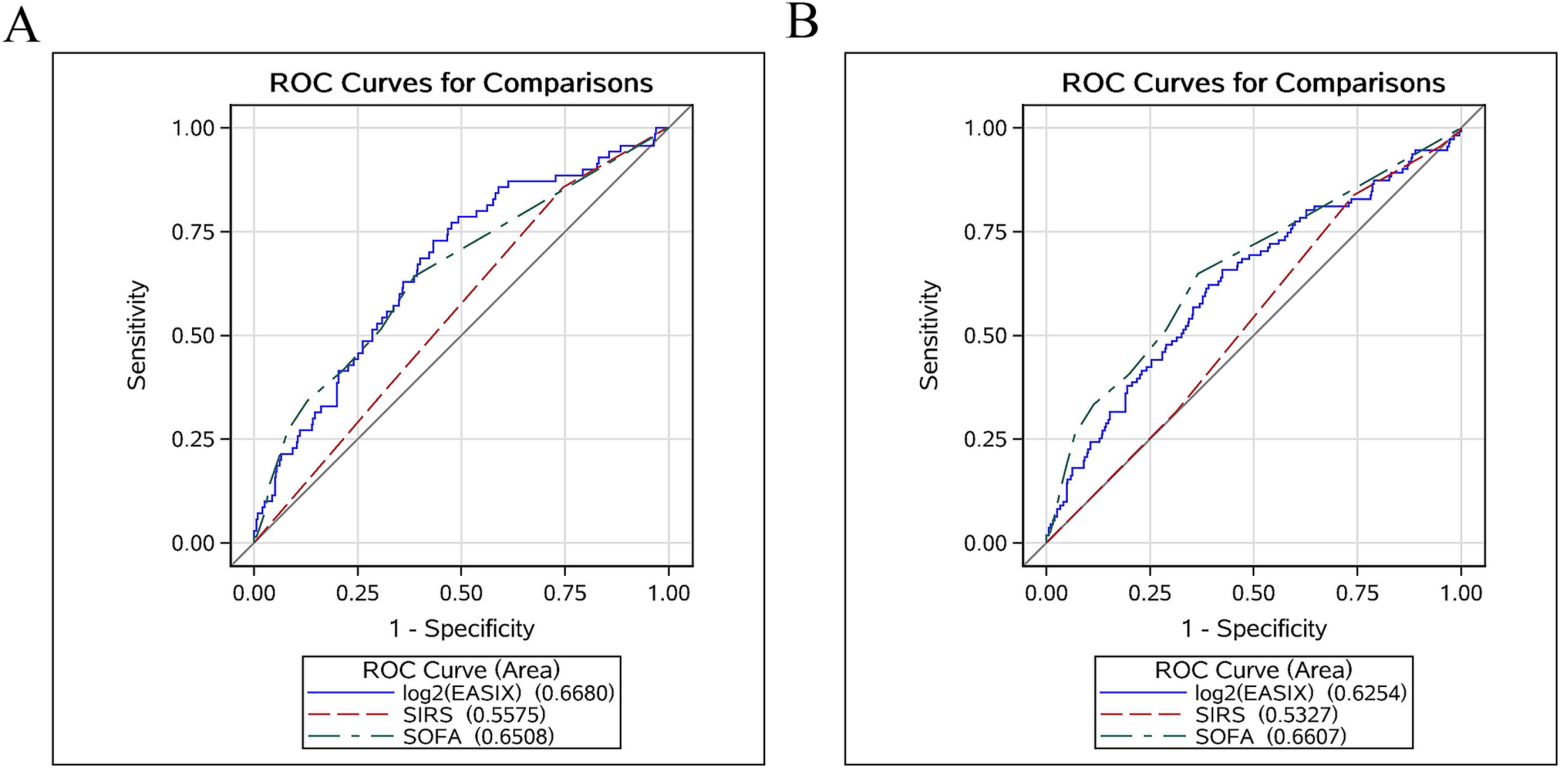
Receiver operator characteristic curves assessing the predictive capability of the log2 (EASIX) index for 28- **(A)** and 90-day **(B)** all-cause mortality (ACM).

ROC curves were plotted for log2 (EASIX), SOFA, and SIRS scores to evaluate their predictive power for 28- and 90-day ACM in patients with AP (Figure 4). Our results indicated that log2 (EASIX) was superior to SOFA and SIRS scores in predicting 28-day ACM in patients with AP in terms of AUC values. Compared with SIRS [55.75% (95% CI: 49.50–61.99)] and SOFA [65.08% (95% CI: 57.97–72.19)], log2 (EASIX) had a significantly higher AUC [66.80% (95% CI: 60.11–73.50)] at day 28 after admission. The predictive efficacy of log2 (EASIX) remained superior to that of SIRS in predicting 90-day ACM, although its AUC was slightly lower than that of SOFA. The ROC curves are presented in Table 4. Our findings demonstrate that log2 (EASIX) is not inferior to traditional indicators for predicting the prognosis of patients with AP.

**Table 4:** Information of ROC curves in Figure 4.

Variables	AUC (%)	95% CI (%)	Threshold	Sensitivity	Specificity
28-day ACM					
log2 (EASIX)	66.80	60.11–73.50	1.13	0.57	0.73
SIRS	55.75	49.50–61.99	2.50	0.26	0.86
SOFA	65.08	57.97–72.19	1.50	0.62	0.64
90-day ACM					
log2 (EASIX)	62.54	56.58–68.49	1.13	0.58	0.66
SIRS	53.27	48.03–58.50	2.50	0.26	0.84
SOFA	66.07	60.31–71.83	1.50	0.64	0.65

### 3.6 Subgroup analysis

**Figure 5:**
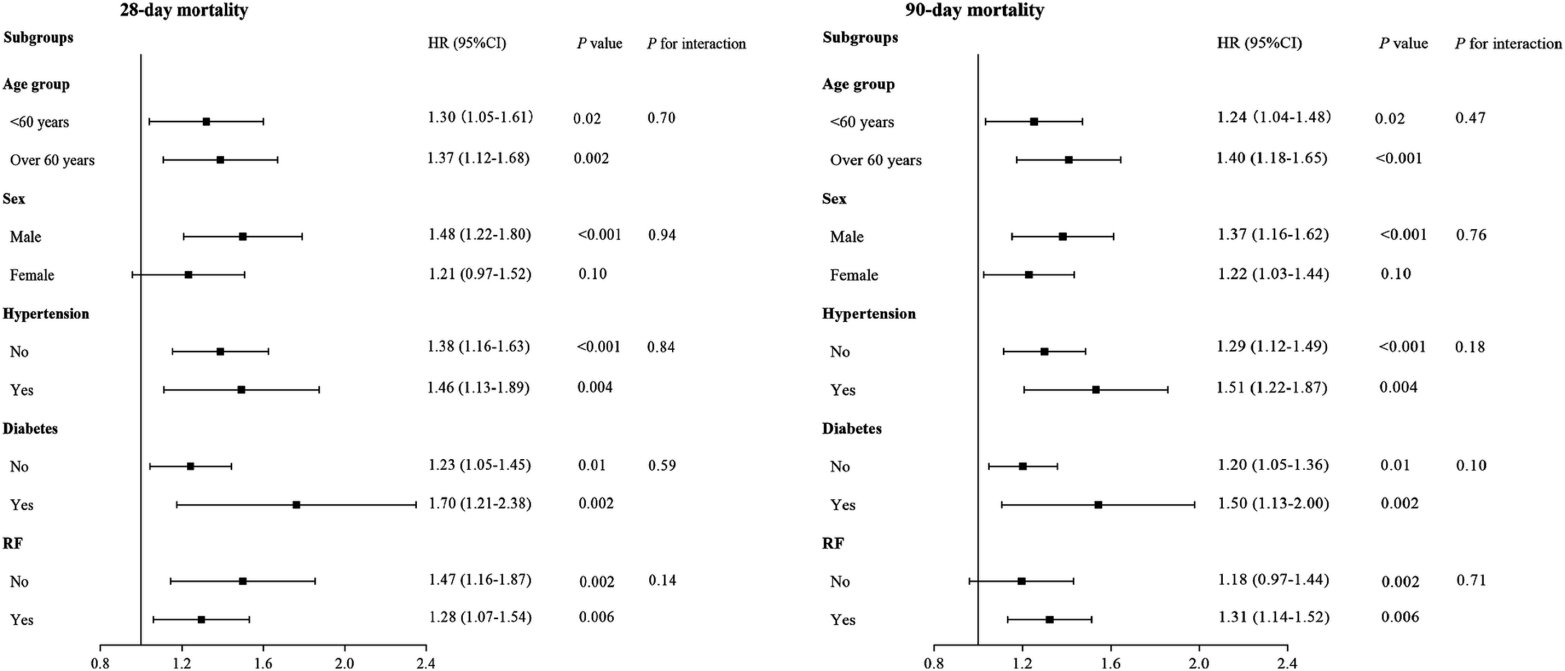
Forest plots of subgroup analyses of the relationship between log2 (EASIX) and 28- and 90-day all-cause mortality (ACM) in patients with acute pancreatitis (AP).

The robustness of the association between log2 (EASIX) and 28- and 90-day ACM was further evaluated through subgroup analyses. When stratified analyses were performed for age, sex, hypertension, diabetes, and RF, there was no significant interaction between log2 (EASIX) and any subgroup (All *p* for interaction >0.05). Further details are presented in Figure 5.

## 4 Discussion

To our knowledge, this study is the first to explore the role of EASIX in predicting the prognosis of AP. Our study demonstrated that elevated EASIX were independently linked to a higher risk of 28- and 90-day ACM in patients with AP. Subgroup analyses confirmed the robustness of these conclusions.

The pathogenesis of AP is a complex process involving several factors, including abnormal activation of digestive enzymes in the pancreas, local or systemic inflammatory responses, tissue necrosis and hemorrhage, and endothelial dysfunction (19, 20). Among these, abnormal activation of digestive enzymes in the pancreas is the core mechanism underlying AP pathogenesis. These enzymes mainly include pancreatic proteases, lipases, and alpha-amylases (21–23). Under normal conditions, they are usually activated for food digestion only after they enter the small intestine. However, in AP, these enzymes are abnormally activated inside the pancreas and digest the pancreatic tissues, leading to necrosis and hemorrhage (24, 25). In addition, activation of these enzymes in the pancreas triggers a strong local or systemic inflammatory response, which exacerbates the damage to the pancreatic tissues and leads to changes such as vasodilatation, increased vascular permeability, and local edema (12).

Recently, the pathophysiological relationship between endothelial dysfunction and AP has attracted increasing attention. When AP occurs, a large number of inflammatory mediators such as tumor necrosis factor-*α*, interleukins (e.g., interleukin-1, interleukin-6), and reactive oxygen species (ROS) are released, and these inflammatory mediators can injure endothelial cells through multiple pathways and trigger endothelial dysfunction (12, 13, 26). Following endothelial cell injury, its barrier function decreases, and vascular permeability increases, leading to edema, hemorrhage, and necrosis of the pancreas and peripancreatic tissues. Endothelial dysfunction leads to microcirculation disorders, manifesting as dysregulated vasoconstriction and dilatation, increased blood viscosity, and microthrombosis (27, 28). Microcirculatory impairment further aggravates pancreatic ischemia and local hypoxia, which promotes pancreatic inflammatory responses and tissue injury. Additionally, during the pathological process of AP, endothelial cells secrete vasoactive substances, including nitric oxide (NO) and endothelin-1 (ET-1) (12, 13), in a dysregulated manner. NO plays vasodilatory, anti-inflammatory, and anti-platelet aggregation roles. Decreased NO synthesis and increased ET-1 levels in AP lead to vasoconstriction and reduced blood flow, which further exacerbates endothelial dysfunction (12, 13). Endothelial cell apoptosis is a crucial factor in the pathogenesis of AP. Inflammatory responses and oxidative stress can lead to endothelial cell apoptosis, resulting in a reduction in endothelial cell numbers and disruption of vascular integrity. Endothelial cell apoptosis also releases more inflammatory mediators and procoagulant factors, further exacerbating inflammation and coagulation (12, 29). Finally, endothelial dysfunction can cause the local inflammatory response to expand into systemic inflammatory response syndrome, which can lead to multi-organ injuries, such as those in the heart, lungs, and kidneys, and increase the risk of morbidity and mortality (12, 26).

EASIX was recently developed to evaluate endothelial cell injury (6). Luft et al. (6) innovatively used three common laboratory indicators (LDH, Crea, and PLT), which are included in the classical diagnostic criteria for thrombotic microangiopathy. They provided robust evidence that EASIX is an effective predictor of overall survival in patients with steroid-refractory graft-versus-host disease following allogeneic stem cell transplantation. In addition, numerous studies have shown that EASIX correlates significantly with the levels of endothelial activation markers, including interleukin-18, CXCL8, CXCL9, insulin-like growth factor-1, tumor inhibitory factor-2, angiopoietin-2, and soluble thrombomodulin (30–33). Subsequently, the prognostic significance of EASIX in other diseases (including low-risk myelodysplastic syndromes, multiple myeloma, neococcal pneumonia, diffuse large B-cell lymphoma, small-cell lung cancer, sepsis, and critically ill patients with advanced liver disease) has also been explored and exploited (7–11).

Of the 620 patients we included, only 97 (15.6%) had a specific etiology documented in detail in their hospitalization data. The etiology of AP was unknown in the remaining 523 patients. EASIX is a surrogate marker for endothelial dysfunction, which is central to the pathophysiology and prognosis of AP regardless of the initial etiology. Therefore, we believe that the etiology of AP may not significantly affect the prognostic value of EASIX for 28- and 90-day ACM in patients with AP.

In this study, we demonstrated that EASIX values were linked to an elevated risk of 28- and 90-day ACM in patients with ICU-managed AP. We believe that this study will help clinicians determine the risk of poor prognosis in patients with AP by assessing EASIX levels. Specifically, we recognize that EASIX, calculated from the first 24 h of hospitalization, could serve as a valuable prognostic tool for identifying high-risk patients who may benefit from more intensive monitoring and timely interventions, such as vasopressor support, mechanical ventilation, or continuous renal replacement therapy. Early risk stratification using EASIX may enable clinicians to prioritize and customize care based on patients’ individual prognoses, thereby improving outcomes in AP management.

The primary strength of this study is that it is the first to investigate the relationship between EASIX and the prognosis of patients with AP. Furthermore, extensive and diverse population data in the MIMIC-IV database allowed us to perform rigorous statistical adjustments to mitigate the effects of potential confounders. Therefore, the robustness of our results was confirmed.

However, our study has some limitations. First, this was a single-center retrospective study, which may limit the generalizability of our findings. Therefore, a multicenter prospective study is necessary to validate our findings. In addition, 620 patients admitted to the ICU for AP were included in this study. Although we are aware of the relatively small number of patients with AP requiring ICU admission, the sample size of this study was relatively small, and larger cohort studies are needed to validate and strengthen our conclusions. Third, the analyses in this study were based on laboratory parameters obtained from the first measurements of patients within 24 h of hospital admission; therefore, it was not possible to assess the dynamic changes in EASIX over time. Dynamic measurement of EASIX is necessary in the future to further elucidate its clinical utility. Fourth, despite our thorough and careful analysis of the participants included in this study, it is worth noting that, because of the limitations of the MIMIC-IV database, the primary populations included in this study were still Americans and other ethnic groups. Therefore, the applicability of our conclusions to other ethnicities and regions requires further investigation. Furthermore, due to limitations within the MIMIC-IV, we were unable to retrieve information on certain other scoring systems commonly used in AP prognosis, such as the Ranson and APACHE II scores. Additionally, because not all patients had complete imaging information, we were unable to analyze the relationship between EASIX and imaging outcomes such as computed tomography severity index. Finally, in performing the statistical analyses, although we adjusted for many potential confounders, we did not completely exclude the influence of other confounders that were not included in the analyses. Future studies should address these limitations to gain more insight into the role of EASIX in predicting the prognosis of patients with AP.

## 5 Conclusion

Elevated EASIX levels are significantly correlated with a higher risk of 28- and 90-day ACM in patients with AP. This finding shows the potential for EASIX to be used by clinicians to determine the risk of poor prognosis in patients with AP. Additional prospective multicenter studies with dynamic EASIX measurements are needed to validate the predictive value of EASIX.

## Data Availability

The original contributions presented in the study are included in the article/supplementary material, further inquiries can be directed to the corresponding author/s.
